# Expanding the application range of the *κ*‑carrageenase OUC-FaKC16A when preparing oligosaccharides from *κ*-carrageenan and furcellaran

**DOI:** 10.1007/s42995-023-00181-2

**Published:** 2023-07-12

**Authors:** Chengcheng Jiang, Francesco Secundo, Xiangzhao Mao

**Affiliations:** 1 College of Food Science and Engineering, Ocean University of China, Qingdao, 266003 China; 2 Laboratory for Marine Drugs and Bioproducts of Qingdao National Laboratory for Marine Science and Technology, Qingdao, 266237 China; 3 Key Laboratory for Biological Processing of Aquatic Products, China National Light Industry, Qingdao, 266237 China; 4 Istituto di Scienze e Tecnologie Chimiche “Giulio Natta”, Consiglio Nazionale delle Ricerche, Via Mario Bianco 9, 20131 Milan, Italy

**Keywords:** Carrageenan oligosaccharides, *κ*-Carrageenase, Expression, Degradation mode, Desulfated oligosaccharides

## Abstract

Carrageenan oligosaccharides are important products that have demonstrated numerous bioactivities useful in the food, medicine, and cosmetics industries. However, the specific structure–function relationships of carrageenan oligosaccharides are not clearly described due to the deficiency of high specific carrageenases. Here, a truncated mutant OUC-FaKC16Q based on the reported *κ*-neocarratetrose (N*κ*4)-producing *κ*-carrageenase OUC-FaKC16A from *Flavobacterium algicola* was constructed and further studied. After truncating the C-terminal Por_Secre_tail (PorS) domain (responsible for substrate binding), the catalytic efficiency and temperature stability decreased to a certain extent. Surprisingly, this truncation also enabled OUC-FaKC16Q to hydrolyze N*κ*4 into *κ*-neocarrabiose (N*κ*2). The offset of Arg_265_ residue in OUC-FaKC16Q may explain this change. Moreover, the high catalytic abilities, the main products, and the degradation modes of OUC-FaKC16A and OUC-FaKC16Q toward furcellaran were also demonstrated. Data suggested OUC-FaKC16A and OUC-FaKC16Q could hydrolyze furcellaran to produce mainly the desulfated oligosaccharides DA-G-(DA-G4S)_2_ and DA-G-DA-G4S, respectively. As a result, the spectrum of products of *κ*-carrageenase OUC-FaKC16A has been fully expanded in this study, indicating its promising potential for application in the biomanufacturing of carrageenan oligosaccharides with specific structures.

**Supplementary Information:**

The online version contains supplementary material available at 10.1007/s42995-023-00181-2.

## Introduction

Carrageenans are natural sulfated polysaccharides that originate from the cell wall of red algae (de Ruiter and Rudolph 1997). These polymers usually consist of D-*β*-galactose (D-Gal, G-unit) and 3,6-anhydro-*α*-D-galactose (D-AHG, DA-unit) (Zhu et al. 2018b). The carrageenan polysaccharide chain has different types of substitutions forming different types of monosaccharide residues, including the C-4 hydroxyl sulfated D-Gal residue (G4S), D-AHG, C-2 hydroxyl sulfated D-AHG residue (DA2S), C-2 hydroxyl sulfated D-Gal residue (G2S), and C-2/C-6 hydroxyl sulfated D-galactose residue (DA2,6-2S) (Kalitnik et al. 2017). Based on the number of sulfate groups, carrageenans are divided into *κ*-carrageenan, *ι*-carrageenan, and *λ*-carrageenan, in which the disaccharide units are G4S-DA, G4S-DA2S, and G2S-DA2,6-2S, respectively (de Ruiter and Rudolph 1997). During past decades, carrageenan has been widely applied in the food industry. Because of its good gel-forming properties (Abraham et al. 2021; Hong et al. 2022; Zia et al. 2017), carrageenan is commonly employed as a gelling agent, thickening agent, or delivery carrier for functional ingredients (Sun et al. 2020; Xie et al. 2019). However, the water-insoluble and macromolecular properties cause their poor bioavailability and bioactivity, which limit further high-value application (Guo et al. 2022; Yu et al. 2002). Low molecular weight (M_W_) carrageenan oligosaccharides have been proved to possess abundant physiological activities, such as antioxidant, antitumor, anti-inflammatory, antivirus, and antibacterial activities (Calvo et al. 2019; Johnson et al. 2021; Kalitnik et al. 2015; Li et al. 2021). Thus, effective methods for preparing carrageenan oligosaccharides are of crucial importance. The enzymatic method based on carrageenase is regarded as an efficient approach for producing carrageenan oligosaccharides under mild conditions (Cao et al. 2021a; Liang et al. 2022). Besides, it has the advantages of obtaining carrageenan oligosaccharides with a specific degree of polymerization (DP) due to the product specificity of carrageenase (Cheong et al. 2018; Guo et al. 2022). Although a considerable number of carrageenases have been identified and studied (Cao et al. 2021b; Zhu et al. 2018b), carrageenases with high specificity are still insufficient to produce carrageenan oligosaccharides and to clarify the structure–function relationship (Zhu et al. 2018b). It has been proved that the bioactivities of carrageenan oligosaccharides are closely related to their structures (Relleve and Abad 2015). Thus, it is essential to explore synthetic routes for the preparations of different and well characterized carrageenan oligosaccharides.

In our previous study, a *κ*-carrageenase OUC-FaKC16A from the marine bacterium *Flavobacterium algicola* was identified, characterized, then assigned to the glycoside hydrolase family 16 (GH16) (Jiang et al. 2022). It is worth noting that OUC-FaKC16A products from degrading *κ*-carrageenan contained ~ 84% *κ*-neocarratetrose (N*κ*4). Among the characterized *κ*-carrageenases (Table 1), it is very rare for a *κ*-carrageenase to produce such a large amount of N*κ*4. Besides, its wide temperature and pH applicability indicated its potential use in the industrial production of *κ*-neocarrageenan oligosaccharides (N*κ*COSs). The OUC-FaKC16A is composed of a GH16 catalytic domain (CD) and a C-terminal Por_Secre_tail (PorS) noncatalytic domain (nonCD). The PorS nonCD was identified to be a C-terminal domain secreted component of the Arg-specific secretion system and adopted an immunoglobulin-like fold (Glew et al. 2012). Until now, the function of PorS domain in glycoside hydrolases has been described only in a GH16 *κ*-carrageenase CgkZ from *Zobellia* sp. ZM-2 (Yu et al. 2017), a GH16 *κ*-carrageenase *κ*-ZL-4 from *Zobellia* sp. ZL-4 (Zhang et al. 2019), and a *β*-agarase AgaM1 from uncultured bacterium (Qu et al. 2020). Therein, it is reported that the specific activity of *κ*-carrageenase *κ*-ZL-4 increased by 1.93 times after truncating its C-terminal carbohydrate binding module family 16 (CBM16) and PorS (Zhang et al. 2019). Moreover, the truncated mutant could produce ten times more *κ*-neocarrabiose (N*κ*2) from hydrolyzing *κ*-carrageenan, although the main products were still N*κ*4. More recently, another nonCD C-terminal immunoglobulin-like domain (Big_2) in *κ*-carrageenase PpCgk was systematically studied indicating that the Big_2 plays a vital role in binding *κ*-carrageenan, affecting specific activity, catalytic efficiency, thermostability, and product component of *κ*-carrageenase PpCgk (Xing et al. 2022). This finding suggests PorS plays a similar function to Big_2 and prompted us to investigate what could be the enzymatic products of OUC-FaKC16A after truncating its PorS.

**Table 1:** *κ*-Carrageenases characterized after 2018

Protein	Bacterial species	GeneBank	Glycoside hydrolase family	Molecular mass (kDa)	Optimal temperature (℃)	Optimal pH	Thermal stability	Hydrolytic products	References
CgkPZ_SP_GH16	*Pseudoalteromonas* sp. ZDY3	QKE61360.1	GH16	32	45	8.0	Stable at 40 °C	N*κ*2, N*κ*4	(Zhao et al. 2021a)
CgkPZ	*Pseudoalteromonas* sp. ZDY3	QKE61360.1	GH16	43	45	8.5	Stable at 40 °C	N*κ*2, N*κ*4	(Zhao et al. 2021a)
CgkZDY3	*Pseudoalteromonas* sp. ZDY3	QKE61360.1	GH16	34	45	8.0	–	N*κ*2, N*κ*4	(Zhao et al. 2021b)
PLJ30	*Pseudoalteromonas carrageenovora* ASY5	–	GH16	–	40–50	6.5–8.0	–	N*κ*2, N*κ*4	(Xiao et al. 2018)
CgkA	*Pedobacter hainanensis* NJ-02	ASA33936.1	GH16	57	40	7.0	Stable at 40 °C	NK4, NK6	(Zhu et al. 2018a)
PpCgkCD	*Pseudoalteromonas porphyrae* LL1	ADD92366.1	GH16	31.5	40	8.0	Stable at 40 °C	N*κ*2, N*κ*4	(Zhao et al. 2018)
CgkA	*Vibrio* sp. SY01	QGN18698.1	GH16	43	40	–	–	N*κ*2 (main), N*κ*4	(Li et al. 2021)
Car1383	Metagenome of Antarctic macroalgae-associated bacteria	–	GH16	40	50	6.0	–	N*κ*2, N*κ*4, N*κ*6	(Li et al. 2022)
*κ*-ZL-4	*Zobellia* sp. ZL-4	–	GH16	–	30	6.0	Stable at 40 °C	N*κ*4 (main), N*κ*6	(Zhang et al. 2019)
*κ*-ZL-4-GH16	*Zobellia* sp. ZL-4	–	GH16	–	55	5.0	Stable at 40 °C	N*κ*2, N*κ*4, N*κ*6	(Zhang et al. 2019)
Car3206	*Polaribacter* sp. NJDZ03	–	GH16	45	55	7.0	Stable at 55 °C	N*κ*2	(Gui et al. 2021)
Cgk16A	*Wenyingzhuangia aestuarii* OF219	AST13124.1	GH16	40.9	25	8.0	–	N*κ*4, N*κ*6	(Shen et al. 2018)
CgkB	*Pedobacter hainanensis* NJ-02	–	GH16	70	40	8.0	–	N*κ*2, N*κ*4, N*κ*6	(Zhu et al. 2018c)
Car19	*Bacillus* sp. HT19	KX184813.1	GH16	45	60	7.0	Stable at 60 °C	N*κ*2	(Li et al. 2019)
OUC-FaKC16A	*Flavobacterium algicola*	MCG9793720.1	GH16	49.6	60	9.0	Stable at 40 °C	N*κ*4 (KC) DA-G-(DA-G4S)_2_ (furcellaran)	This study
OUC-FaKC16Q	*Flavobacterium algicola*	MCG9793720.1	GH16	40.1	60	7.0	Stable at 40 °C	N*κ*2 (KC) DA-G-(DA-G4S) (furcellaran)	This study

Here, the PorS-truncated mutant OUC-FaKC16Q and mutant FaPorS, only containing the PorS nonCD, were constructed and expressed for investigating the role of PorS in *κ*-carrageenase OUC-FaKC16A. The catalytic efficiency, thermal stability, and degradation mode of OUC-FaKC16A and OUC-FaKC16Q were further studied and compared. Intriguingly, the final products of OUC-FaKC16Q were N*κ*2 instead of N*κ*4. Moreover, another hybrid *β*/*κ*-carrageenan furcellaran was used as the substrate to explore their different enzymatic products and degradation mode. As a result, the application potential of *κ*-carrageenase OUC-FaKC16A for preparing oligosaccharides with specific DP and structure was fully highlighted in this study.

## Results

### The influence on hydrolytic activity of OUC-FaKC16A by noncatalytic FaPorS domain

**Fig. 1:**
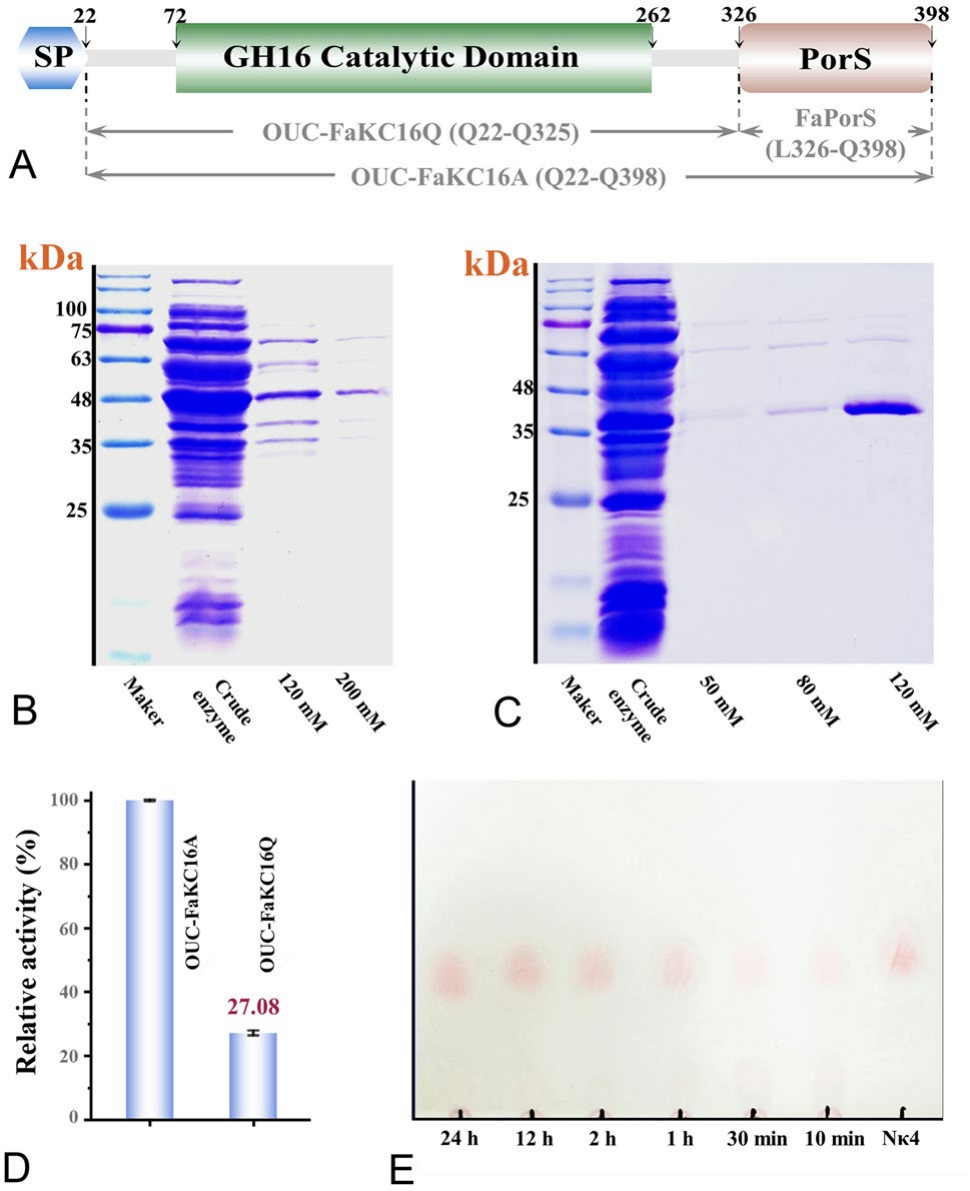
**A** Conserved domains diagram of OUC-FaKC16A. SDS-PAGE of pure enzymes **B** OUC-FaKC16A and **C** OUC-FaKC16Q. **D** Relative activities of OUC-FaKC16Q compared with OUC-FaKC16A. **E** TLC analysis of the hydrolysis of *κ*-carrageenan with OUC-FaKC16A

To investigate the effects of PorS nonCD on the hydrolytic activity and enzymatic properties of OUC-FaKC16A, the gene fragments encoding OUC-FaKC16A (Q22-Q398), OUC-FaKC16Q (Q22-Q325), and FaPorS (L326-Q398) (Fig. 1A) were cloned into plasmid pCold (modified from pColdSUMO) and expressed in an *E*. *coli* RTS(DE3) host. After induction, the crude enzymes were purified by an NTA column and the purity of OUC-FaKC16A and OUC-FaKC16Q was analyzed by SDS-PAGE. The predicted M_W_s of OUC-FaKC16A, OUC-FaKC16Q, and FaPorS were 49.6, 40.1, and 11.1 kDa, respectively, in agreement with the sodium dodecyl sulfate–polyacrylamide gel electrophoresis (SDS-PAGE) results (Fig. 1B, C, and Supplementary Fig. S1A). Concerning the hydrolytic activity of OUC-FaKC16Q toward *κ*-carrageenan, it resulted in 27% compared to that of OUC-FaKC16A (Fig. 1D). FaPorS exhibited no hydrolytic activity (data not shown).

SDS-PAGE was performed to distinguish the proteins that co-precipitate with the polysaccharides remaining in the supernatant. The results suggested that FaPorS is the main component in polysaccharide precipitation (Supplementary Fig. S1B), implying its ability to specifically bind *κ*-carrageenan. Moreover, scanning electron microscopy (SEM) analysis allowed the observation of the surfaces of *κ*-carrageenan after FaPorS treatment. As shown in Supplementary Fig. S2A, the *κ*-carrageenan surface was regular and smooth if treated with bovine serum albumin (BSA), while it was irregular and porous when treated with FaPorS (Supplementary Fig. S2B), suggesting FaPorS binds to the *κ*-carrageenan surface altering its regular structure. Similar functions have also been shown in noncatalytic Big_2 domain from *κ*-carrageenase PpCgk (Xing et al. 2022).

The phylogenetic tree of the noncatalytic domains of PorS and Big_2 from different bacteria suggested they belong to different evolutionary branches. All *κ*-carrageenases and other hydrolases (including *β*-agarase, α-amylase, pectate lyase, cellulase, and peptidase) contained only one PorS domain, strictly preserved in their C-terminus, similar to the characteristics of Big_2 domain (Xing et al. 2022). The sequence alignment of *κ*-carrageenase with a PorS domain was further performed to analyze the conserved region. As shown in Supplementary Fig. S3B, the sequences encoding four *β*-strands in all PorS domains exhibited high homology, implying such *β*-strands may play an important role in substrate binding. In addition, the positively charged amino acids (Arg and Lys) are crucial in Big_2 domains to bind *κ*-carrageenan (Xing et al. 2022). In *κ*-carrageenase endowed of a PorS domain, the positively charged amino acids are ~ 10%, which is close to those of other hydrolases containing the PorS domain (Supplementary Fig. S3C) and to that of the *κ*-carrageenase with the Big_2 domain (Xing et al. 2022). This confirms the substrate-binding function of the PorS domain either in *κ*-carrageenase or in other hydrolases (including *β*-agarase, α-amylase, pectate lyase, cellulase, and peptidase).

### Biochemical characterization of OUC-FaKC16Q

**Fig. 2:**
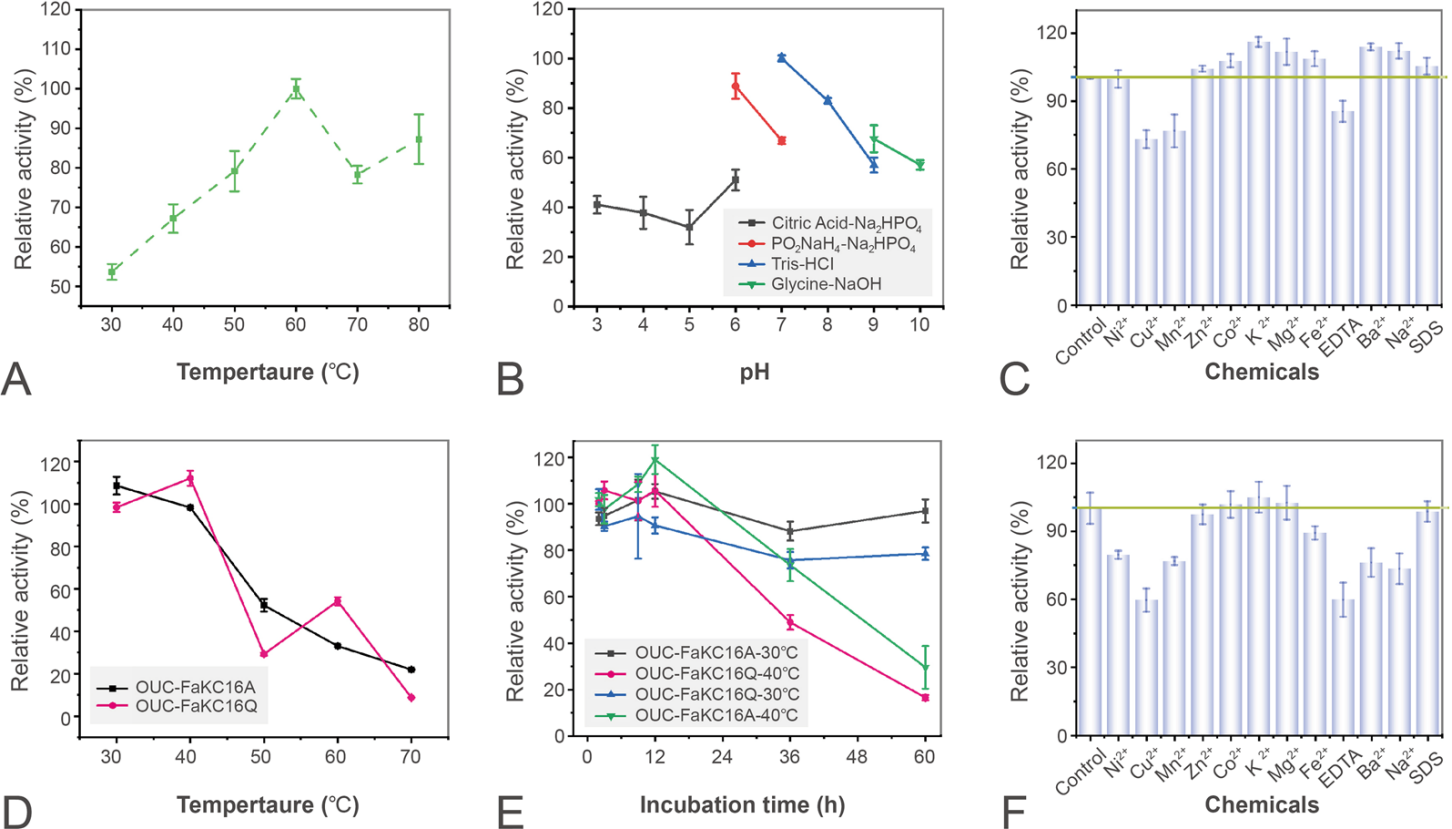
Characterization of OUC-FaKC16Q. **A** Effects of temperature on the activity of OUC-FaKC16Q. **B** Effects of pH on the activity of OUC-FaKC16Q. Effects of metal ions and chemicals on the enzyme activities of **C** OUC-FaKC16A and **F** OUC-FaKC16Q. Thermostability of the OUC-FaKC16A and OUC-FaKC16Q by incubating **D** at 30, 40, 50, 60, and 70 ℃ for 1 h and **E** at 30 and 40 °C for 60 h. All measurements were performed in triplicate, error bars indicate standard deviation of measurement

The optimum temperature of OUC-FaKC16Q was 60 ℃ in accordance with OUC-FaKC16A (Fig. 2A). The optimum pH was 7.0, which is different from that of OUC-FaKC16A in which the optimum pH value is 9.0 (Fig. 2B). Moreover, while the OUC-FaKC16A shows more than 80% relative activity in a wide pH range from 4.0 to 9.0. This range was only from 6.0 to 8.0 in the case of OUC-FaKC16Q (Fig. 2B).

Concerning thermal stability, after 1 h incubation, both enzymes showed good stability at 30 ℃ and 40 ℃ but poor stability above 40 °C (Fig. 2D). Nevertheless, when incubated for a longer time, OUC-FaKC16A still retained ~ 70% of its initial activity after incubating for 36 h at 40 °C while OUC-FaKC16Q only retained ~ 50% (Fig. 2E), implying deletion of FaPorS causes a decrease in OUC-FaKC16A thermostability.

The effects of metal ions and chemicals toward hydrolytic activity are shown in Fig. 2C. Cu^2+^, Mn^2+^. EDTA inhibit the OUC-FaKC16A’s hydrolytic activity and also plays the same inhibition role in OUC-FaKC16Q. The hydrolytic activity of this latter one undergoes inhibition in the presence of Ni^2+^, Ba^2+^, and Na^+^ as well (Fig. 2F). Therefore, the tolerance of OUC-FaKC16A toward metal ions is weakened after truncation of its PorS nonCD.

The comparison of the kinetic constants indicated *K*m and *k*cat were 8.54 mg/mL and 1.52 s^−1^ for OUC-FaKC16A, and 12.43 mg/mL and 0.53 s^−1^ in the case of OUC-FaKC16Q, revealing FaPorS plays a vital role in maintaining the high substrate affinity and catalytic ability of OUC-FaKC16A.

### Degradation modes of *κ*-carrageenan with OUC-FaKC16Q

**Fig. 3:**
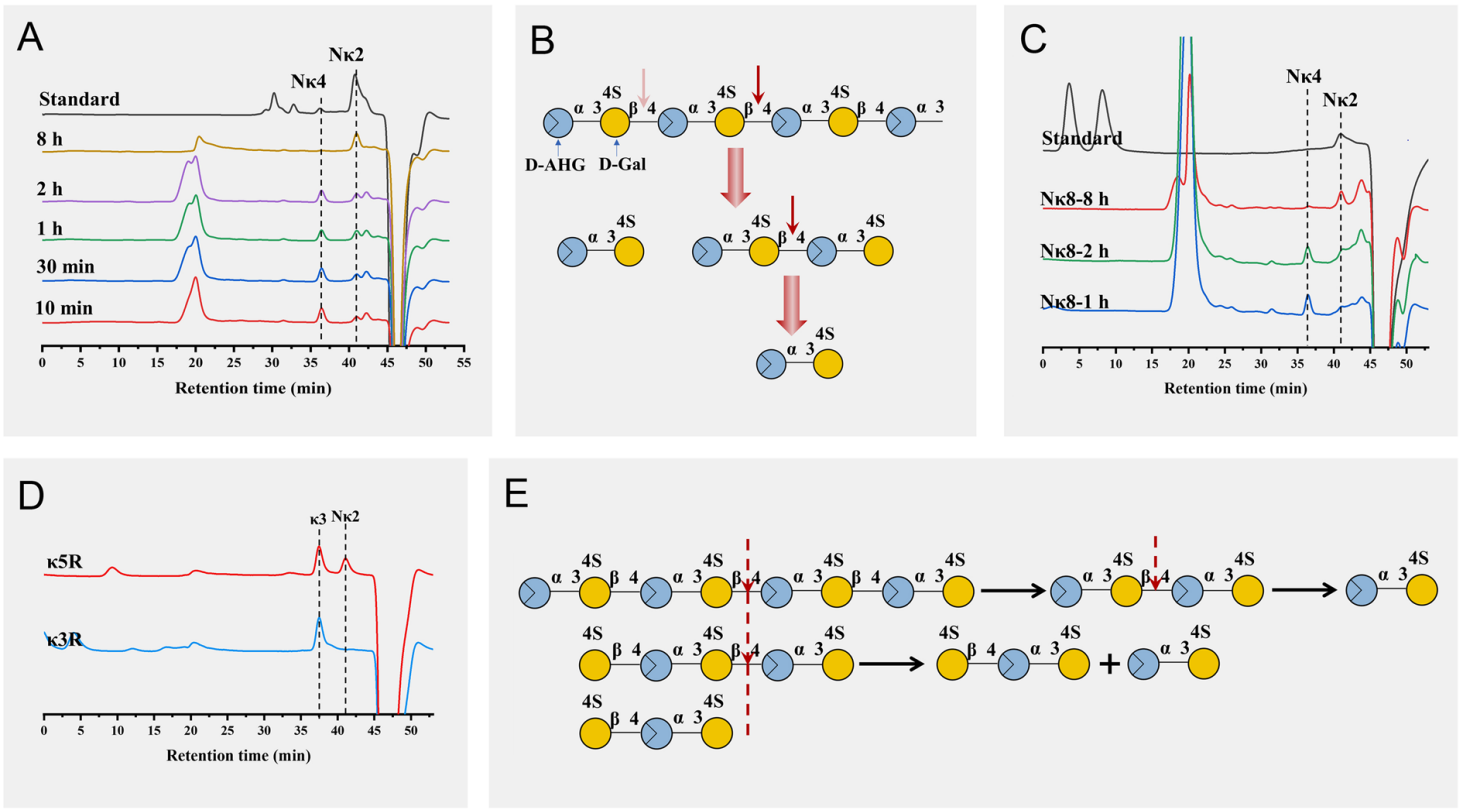
HPLC analysis of degradation mode of OUC-FaKC16Q toward (**A**) *κ*-carrageenan, **C** *κ*-neocarraoctaose (N*κ*8), **D** *κ*-carratriose (*κ*3), and *κ*-carrapentaose (*κ*5). Schematic diagram of OUC-FaKC16Q acting on (**B**) *κ*-carrageenan and (**E**) oligosaccharides substrates. Blue and red circles represent D-AHG and D-Gal monomers, respectively

The hydrolysis products of OUC-FaKC16Q, carried out and monitored by high-performance liquid chromatography (HPLC), showed that N*κ*2 and N*κ*4 were formed within 10 min. The amount of N*κ*2 gradually increased and N*κ*4 decreased. After 8 h, the reaction products only contained N*κ*2 (Fig. 3A). This outcome is different from OUC-FaKC16A, which only produced N*κ*4 as the final product (Fig. 1E). Thus, OUC-FaKC16Q shows a novel hydrolytic capacity toward N*κ*4, converting N*κ*4 into N*κ*2 with the reaction progress (Fig. 3B).

The degradation modes of oligosaccharides by OUC-FaKC16Q were explored also using *κ*-neocarraoctaose (N*κ*8) as the starting substrate, which was initially transformed mainly into N*κ*4 and, to a lesser extent, N*κ*2. N*κ*4 was then completely transformed into N*κ*2 in 8 h (Fig. 3C). In addition, the *κ*-carratriose (*κ*3) and *κ*-carrapentaose (*κ*5) with a non-reducing end of G4S were also used as substrates. As shown in Fig. 3D, the *κ*5 could be hydrolyzed by OUC-FaKC16Q to form N*κ*2 and *κ*3, while *κ*3 was not hydrolyzed. We concluded that the minimum cutting units of OUC-FaKC16Q were N*κ*2 and *κ*3, and the shortest oligosaccharide substrate was N*κ*4 (Fig. 3E). These findings suggest that the deficiency of FaPorS enabled OUC-FaKC16A to convert N*κ*4 into N*κ*2. It is reported that the mutant of *κ*-carrageenase *κ*-ZL-4, after deleting CBM16 and PorS, could produce ten times more N*κ*2, but the main products were still N*κ*4 and *κ*-neocarrahexaose (N*κ*6) (Zhang et al. 2019).

### Molecular docking to explore different hydrolysis abilities toward N*κ*4 of OUC-FaKC16A and OUC-FaKC16Q

To shed light on the catalytic behaviors of OUC-FaKC16A and OUC-FaKC16Q toward N*κ*4, their three-dimensional structures were predicted by the online tool Phyre2 via Intensive modeling mode (Kelley et al. 2015). Simulated structures were evaluated by VERIFY3D (Eisenberg et al. 1997), suggesting that 81.2% and 81.9% of residues in OUC-FaKC16A and OUC-FaKC16Q (Supplementary Fig. S4), respectively, averaged a 3D-1D score, with ≥ 0.2 indicating their reliability. As shown in Supplementary Fig. S5A, the overall structures of OUC-FaKC16A and OUC-FaKC16Q were similar, except for the FaPorS domain, which is absent in OUC-FaKC16Q. It is noteworthy that OUC-FaKC16Q tends to form a smaller substrate-binding channel than OUC-FaKC16A (Supplementary Fig. S5B), suggesting this feature is useful to catalyze the hydrolysis of shorter chain oligosaccharides.

**Fig. 4:**
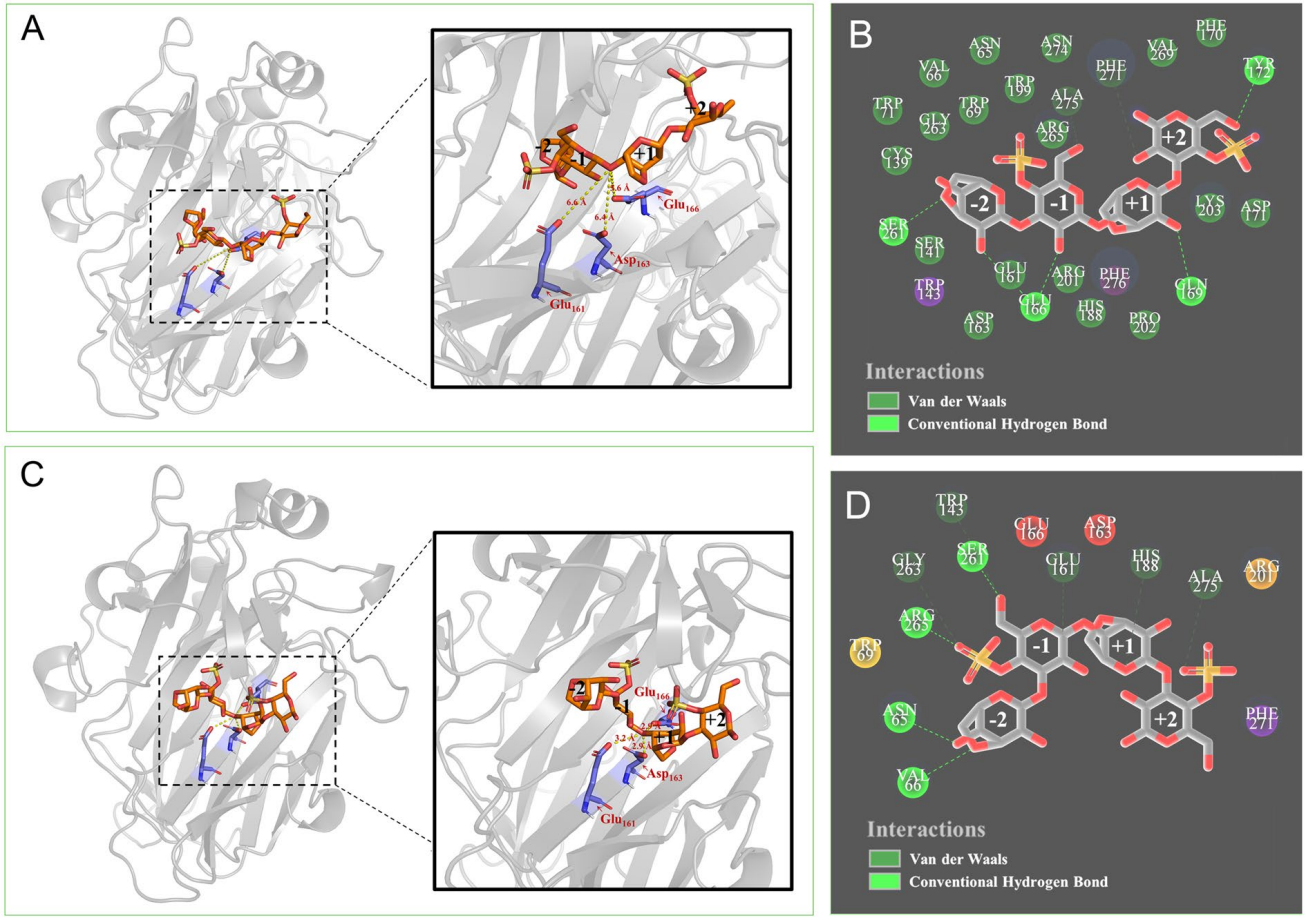
**A** Molecular docking result of OUC-FaKC16A with N*κ*4 and **B** its 2D diagram of receptor–ligand interactions. **C** Molecular docking result of OUC-FaKC16A with *κ*-neocarratetrose (N*κ*4) and **D** its 2D diagram of receptor–ligand interactions

Molecular docking of OUC-FaKC16A and OUC-FaKC16Q with N*κ*4 was further performed. In the docking result of OUC-FaKC16A, the distances from the catalytic residues Glu_161_, Asp_163_, and Glu_166_ to the *β*-1,4-glycosidic linkage between -1 and + 1 subsites were 6.6, 6.4 and 5.6 Å, respectively (Fig. 4A). Instead, in the docking result of OUC-FaKC16Q, these distances were 3.2, 2.9 and 2.9 Å, respectively (Fig. 4B), indicating binding of N*κ*4 with OUC-FaKC16Q is more likely and stable than with OUC-FaKC16A, providing a possible explanation to the catalytic behavior of OUC-FaKC16Q towards N*κ*4.

**Fig. 5:**
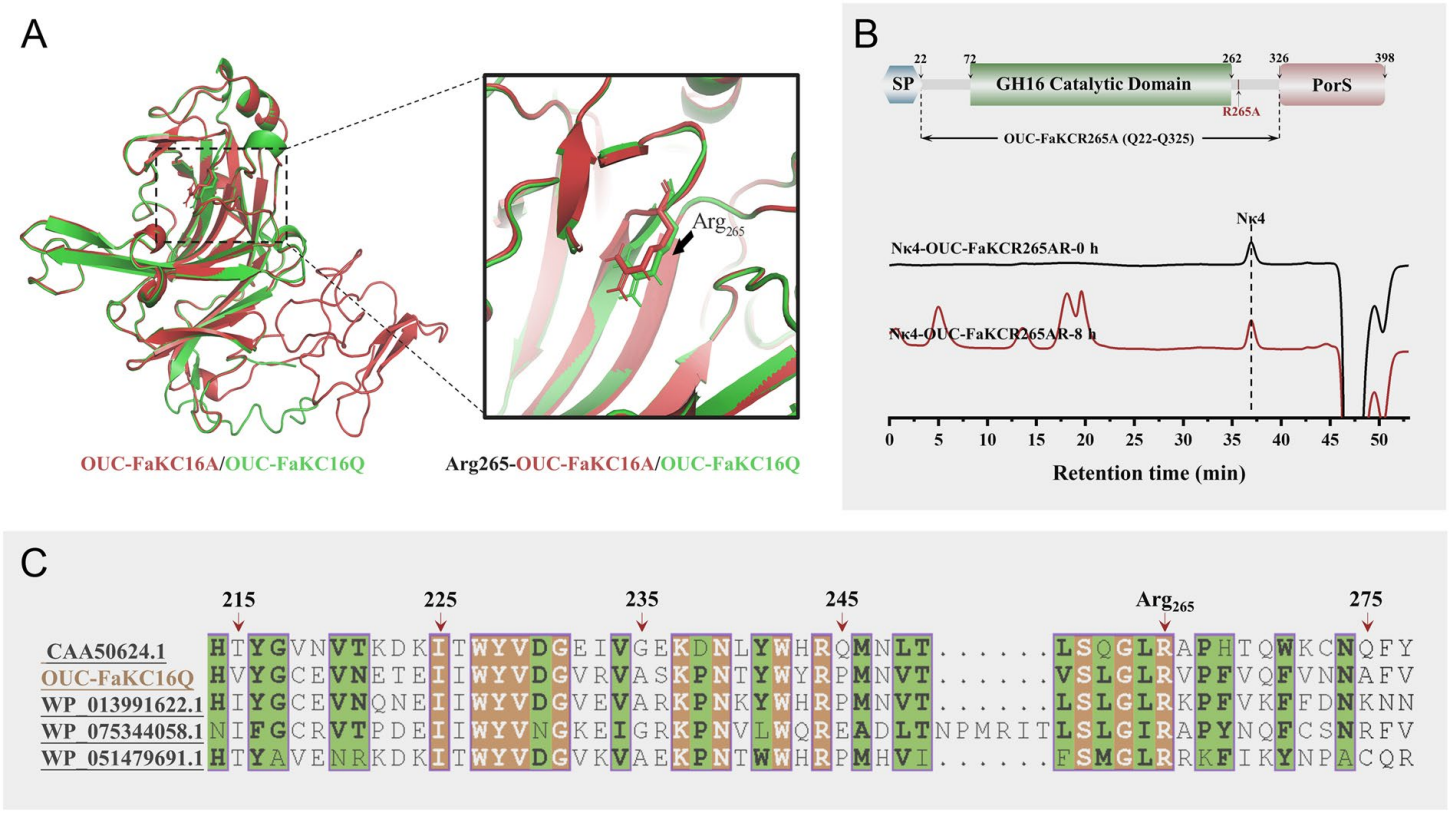
**A** Comparison of the three-dimensional structure of OUC-FaKC16A and OUC-FaKC16Q. **B** HPLC analysis of the hydrolytic activity of OUC-FaKCR265A toward *κ*-neocarratetrose (N*κ*4). **C** Sequence alignment of GH16 *κ*-carrageenases and of Arg_265_ residue

Besides, it is noteworthy that the Arg_265_ residue of OUC-FaKC16Q formed a hydrogen bond with − 1 subsite’s G4S unit (Fig. 4D), but without the same hydrogen bond formed by Arg_265_ residue with − 1 subsite presence in OUC-FaCK16A’s docking conformation (Fig. 4B). We further compared the positions of Arg_265_ residue in OUC-FaKC16A and OUC-FaKC16Q. As shown in Fig. 5A, the position of Arg_265_ in OUC-FaKC16Q is closer to the active sites. Sequence alignment shows that Arg_265_ residue of OUC-FaKC16A corresponds to Arg_260_ residue of *κ*-carrageenase PcCgkA (Fig. 5C), identified as the key residue of binding − 1 site’s G4S unit allowing for the production of N*κ*4 from hydrolyzing *κ*-carrageenan (Matard-Mann et al. 2017). The mutant OUC-FaKC16QR265A, constructed based on OUC-FaKC16Q and used for transforming N*κ*4, did not produce any N*κ*2 after an 8 h reaction, implying the crucial role of Arg_265_ residue in the degradation of N*κ*4 (Fig. 5B).

### Degradation modes of OUCFaKC16A and OUC-FaKC16Q toward furcellaran

**Fig. 6:**
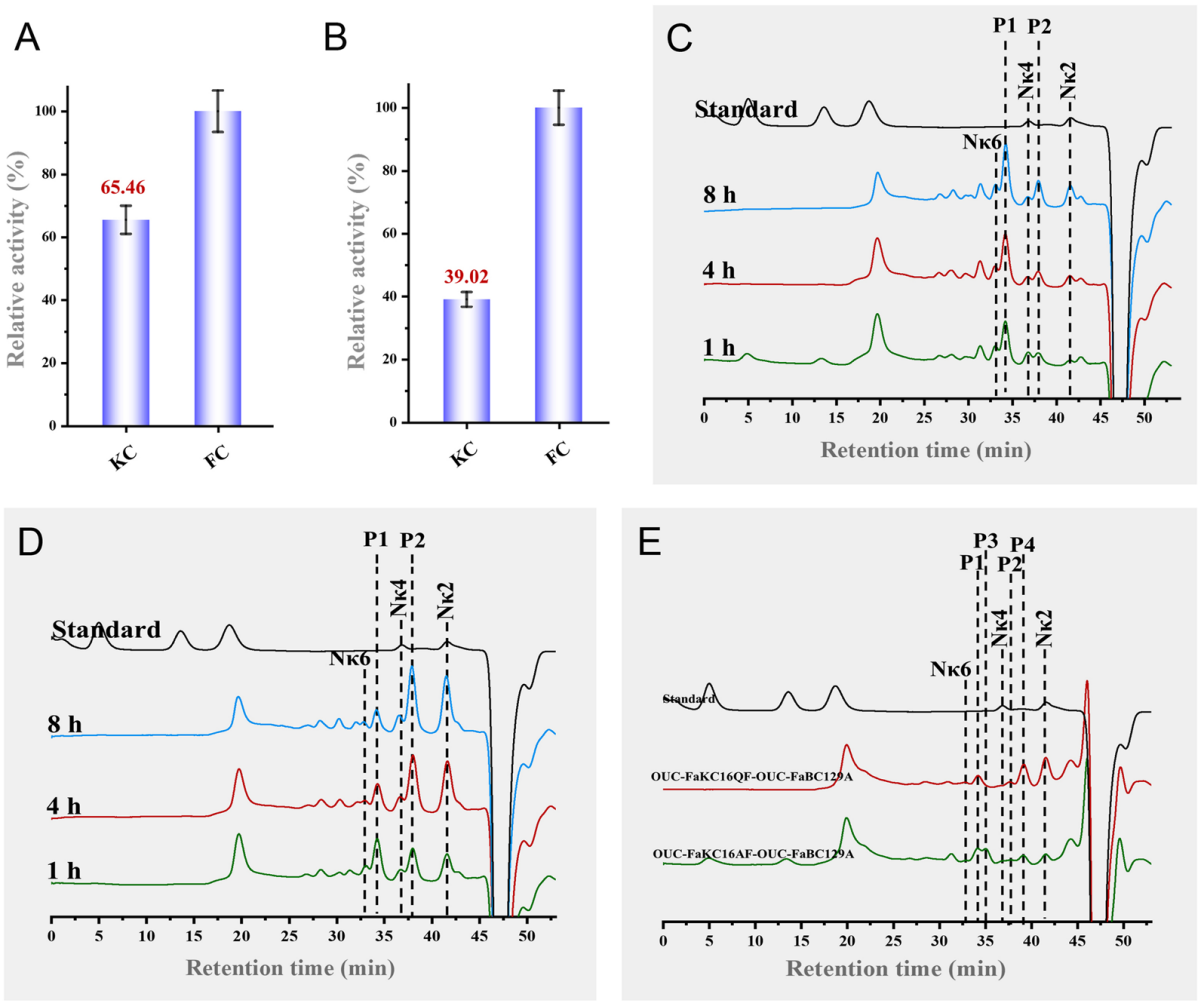
Hydrolytic effects of **A** OUC-FaKC16A and **B** OUC-FaKC16Q toward *κ*-carrageenan (KC) and furcellaran (FC). HPLC analyses of degradation modes of **C** OUC-FaKC16A and **D** OUC-FaKC16Q toward FC. **E** HPLC analyses of products from hydrolyzing the FC hydrolysates of OUC-FaKC16A and OUC-FaKC16Q using the exo-α-3,6-anhydro-D-galactosidase OUC-FaBC129A

**Fig. 7:**
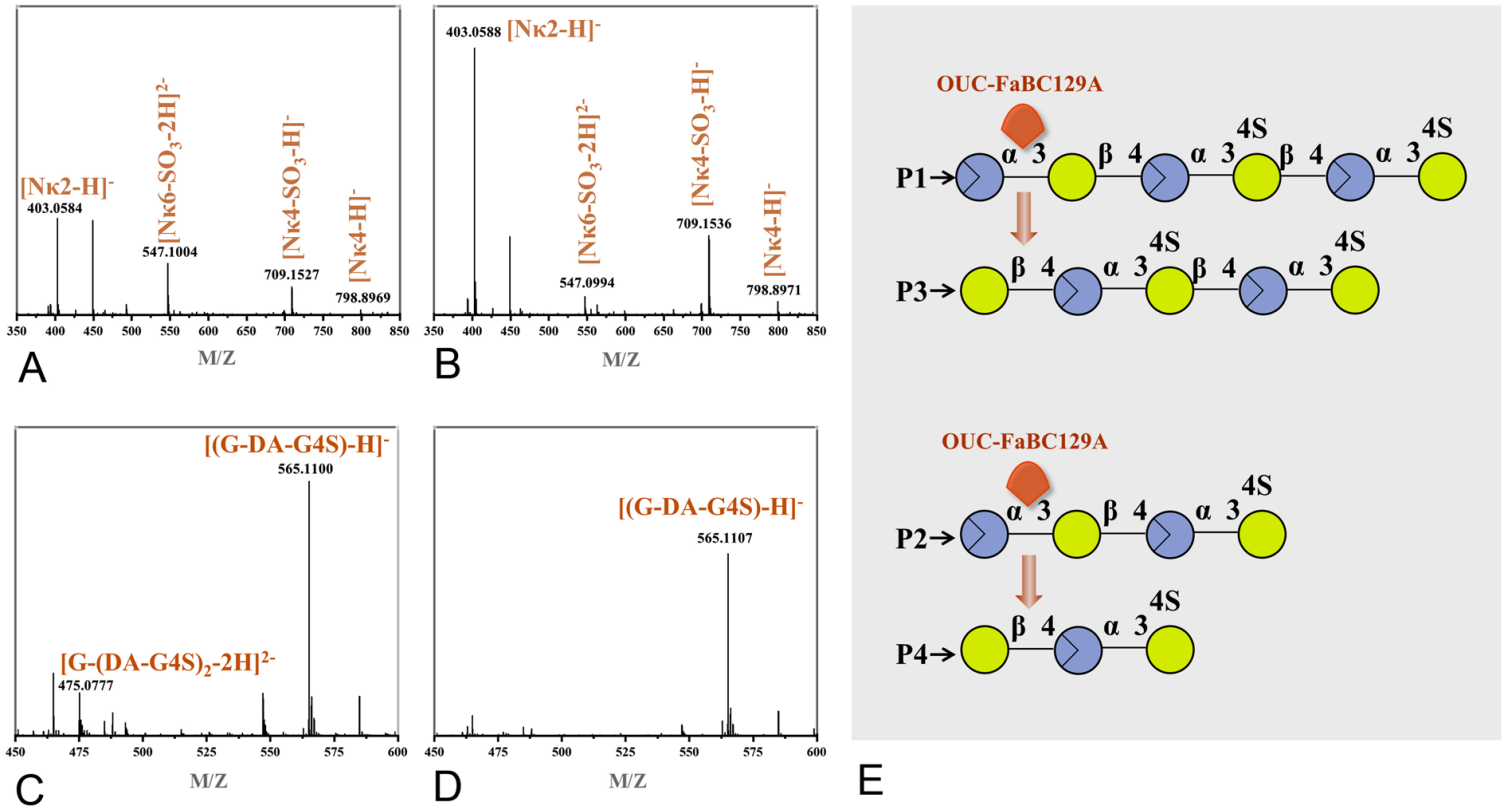
MS analyses of degradation products of **A** OUC-FaKC16A and **B** OUC-FaKC16Q toward furcellaran. MS analyses of products from hydrolyzing the furcellaran hydrolysates of **C** OUC-FaKC16A and **D** OUC-FaKC16Q using the exo-α-3,6-anhydro-D-galactosidase OUC-FaBC129A. **E** Schematic diagram of O. Blue and red circles represent D-AHG and D-Gal monomers, respectively

The degradation products and modes of OUC-FaKC16A and OUC-FaKC16Q were tested on furcellaran to expand their application range. Using the 3,5-dinitrosalicylic acid (DNS) method, both enzymes showed better hydrolase activity toward furcellaran than *κ*-carrageenan. The hydrolytic efficiency of OUC-FaKC16A and OUC-FaKC16Q toward furcellaran was 1.52 and 2.56 times higher than with *κ*-carrageenan as substrate, respectively (Fig. 6A, B). The kinetic of the OUC-FaKC16A-mediated degradation process monitored with HPLC analysis showed the accumulation of N*κ*2, P2, P1, and N*κ*6 as the reaction proceeded, with P1 as the main product (Fig. 6C). Instead, in the OUC-FaKC16Q-mediated degradation process, after 1 h, the products were N*κ*2, P2, N*κ*4, P1, and N*κ*6. The P1 decreased during the reaction, while the P2 and N*κ*2 increased becoming the main components after 8 h of reaction (Fig. 6D). In addition, LC–mass spectra (LC–MS) was performed to analyze the 8 h products. The data suggest the M_W_s of P1 and P2 have one less sulfate group than *κ*-neocarrahexaose (N*κ*6) and N*κ*4, respectively (Supplementary Fig. S6). MS analysis also suggested this conclusion (Fig. 7A, B), indicating that the desulfated N*κ*COSs with one sulfate group removal can be obtained from hydrolyzing furcellaran by OUC-FaKC16A and OUC-FaKC16Q.

To explore from which position in P1 and P2 the sulfate group is removed, an exo-*α*-3,6-anhydro-D-galactosidase (ADAG) OUC-FaBC129A was used. OUC-FaBC129A can act on the first *α*-1,3-glycosidic linkage from the non-reducing end of neocarrageenan oligosaccharides (NCOSs) to release D-AHG, but the NCOSs should be a *β*-neocarrabiose (N*β*2) motif in their non-reducing end (Ficko-Blean et al. 2017; Jiang et al. 2022). HPLC showed that P4 and P3 were produced from P2 and P1 after hydrolysis with OUC-FaBC129A, respectively (Fig. 6E). The comparison of the MS analyses indicates P3 and P4 should be oligosaccharides with DPs of 5 and 3, respectively (Fig. 7C, D). Thus, the structures of P1 and P2 should be DA-G-(DA-G4S)_2_ and DA-G-DA-G4S, respectively, because they can be hydrolyzed with ADAG OUC-FaBC129A (Fig. 7E). Correspondingly, the structures of P3 and P4 were G-(DA-G4S)_2_ and DA-G-DA-G4S, respectively (Fig. 7E). The above results highlight that the OUC-FaKC16A and OUC-FaKC16Q can hydrolyze furcellaran preparing desulfated N*κ*COSs. Meaningfully, their main hydrolysates are different, DA-G-(DA-G4S)_2_ and DA-G-DA-G4S for OUC-FaKC16A and OUC-FaKC16Q, respectively.

## Discussion

Previously characterized GH16 *κ*-carrageenase OUC-FaKC16A exhibited high product specificity capable of producing ~ 84% N*κ*4 from hydrolyzing *κ*-carrageenan. Similar to the influence of CBM and PorS in *κ*-carrageenases *κ*-ZL-4 (Zhang et al. 2019) and ZgCgkA (Matard-Mann et al. 2017), of PorS in *β*-agarase rAgaM1 (Qu et al. 2020), and Big_2 in *κ*-carrageenase PpCgk (Xing et al. 2022), functional domain analysis showed OUC-FaKC16A contained a C-terminal PorS nonCD in which truncation can modify the catalytic activity and products composition of hydrolysis. However, the sole influence of PorS in *κ*-carrageenase has not yet been elucidated. The mutant OUC-FaKC16Q had an ~ 83% decrease in its specific activity compared with OUC-FaKC16A. To the contrary, in another study, the loss of the PorS of the *κ*-carrageenase CgkZ from *Zobellia* sp. ZM-2 caused an improvement of its specific activity, expressed as U/mg (Yu et al. 2017). After conversion with U/μmol as enzyme activity unit, the specific activity of the truncated mutant cgkZ∆Pst was 2.2 × 10^5^ U/μmol, while wild-type cgkZ was 2.3 × 10^5^ U/μmol, indicating PorS might play a different role in keeping the high enzymatic activity in different *κ*-carrageenases. Most recently, the noncatalytic Pig_2 domain in *κ*-carrageenase PpCgk from *Pseudoalteromonas porphyrae* LL1 has been proved to be crucial for the specific binding and breaking of *κ*-carrageenan (Xing et al. 2022). It is speculated that the noncatalytic PorS domain in OUC-FaKC16A may play the same roles of Pig_2 domain. The results of SDS-PAGE (Supplementary Fig. S1) detecting bound ProS protein in *κ*-carrageenan precipitation and SEM (Supplementary Fig. S2) of observing PorS-treated *κ*-carrageenan help demonstrate that PorS can bind *κ*-carrageenan and break its regular structure.

Molecular characterization results suggest that the pH applicability, thermostability, and metal ion resistance of OUC-FaKC16Q decrease compared with wild-type OUC-FaKC16A. These results are similar to changes caused by deletion of the Big_2 nonCD in *κ*-carrageenase PpCgk (Xing et al. 2022), which indicated that PorS and Big_2 nonCD have similar functions for binding *κ*-carrageenan. Further degradation mode analysis revealed that the final hydrolysis product of *κ*-carrageenan with OUC-FaKC16A after truncating the PorS nonCD is N*κ*2 instead of N*κ*4. The importance that N*κ*2-producing *κ*-carrageenase OUC-FaKC16Q was obtained lies in the fact that characterized *κ*-carrageenases with a single product are relatively rare (Table 1) (Zhu et al. 2018b). Only *κ*-carrageenases Car19 and Car3206 can hydrolyze *κ*-carrageenan to produce single N*κ*2 but with an exolytic action mode (Gui et al. 2021; Li et al. 2019). Thus, the different OUC-FaCK16Q mode of hydrolysis herein shown brings new insight into the polysaccharide degradation mode of *κ*-carrageenase.

While exploring the influence of the truncation of C-terminal CBM and PorS nonCD in *κ*-carrageenase *κ*-ZL-4, Zhang et al. (2019) observed that the truncated mutant *κ*-ZL-4-GH16 produced 24.5%N*κ*2 as the final hydrolysis product, while the wild-type *κ*-ZL-4 almost did not produce N*κ*2 from degrading *κ*-carrageenan. A comparison of their predicted three-dimensional structures revealed that *κ*-ZL-4-GH16 formed a closed catalytic tunnel and *κ*-ZL-4 formed a slightly open tunnel, potentially explaining the catalytic N*κ*2 production of *κ*-ZL-4-GH16. In particular, the molecular docking of *κ*-ZL-4-GH16 and *κ*-ZL-4 with N*κ*6 showed that the catalytic residue Asp_164_ formed a smaller distance with the + 2 subsite’s D-Gal residue in the case of *κ*-ZL-4-GH16, facilitating hydrolysis of N*κ*6 to N*κ*2 (Zhang et al. 2019). Similar to this study, the three-dimensional structures of wild-type OUC-FaKC16A and truncated mutant OUC-FaKC16Q were simulated and compared. Although both shaped the partially closed catalytic tunnel, the channel in OUC-FaKC16Q was smaller, indicating it could bind smaller oligosaccharide ligands. Further molecular docking of OUC-FaKC16A and OUC-FaKC16Q with N*κ*4 suggested the catalytic residues Glu_161_, Asp_163_, and Glu_166_ of OUC-FaKC16Q interacted with N*κ*4 in the smaller distances. Also, in the docking conformation of OUC-FaKC16Q, there are four residues (Asn_65_, Val_66_, Ser_261_, and Arg_265_) forming hydrogen bonds with the -2 or -1 subsites (Fig. 4D). Only two residues (Ser_261_ and Glu_166_) formed hydrogen bonds in the case of OUC-FaKC16A (Fig. 4B). Additionally, the C-terminal Big_2 domain also demonstrated the influence in modulating the action mode of *κ*-carrageenase PpCgk whose main hydrolysate is N*κ*4. The data suggested that the mutant PpCgkCD after truncated Big_2 domain would produce 1.5 times of N*κ*2 compared with wild-type PpCgk. This phenomenon was interpreted as the carrageenan-binding capacity of Big_2 domain makes it more difficult for the catalytic module to separate from the substrate after completion of one hydrolysis, resulting in less N*κ*2 products in the hydrolysate of wild-type PpCgk (Xing et al. 2022). The FaPorS nonCD of OUC-FaKC16A also proved the function of binding *κ*-carrageenan, thus causing the change of OUC-FaKC16A’s product after truncating it.

In previous research, the *κ*-carrageenase from *Pseudoalteromonas carrageenovora* (Michel et al. 2001) had been used to hydrolyze furcellaran to obtain oligosaccharides (Ficko-Blean et al. 2017), revealing the catalytic effects of *κ*-carrageenase toward furcellaran, though specific product composition was not given. OUC-FaKC16A and OUC-FaKC16Q can obtain different final products from the hydrolysis of *κ*-carrageenan, prompting us to investigate whether they can also obtain different products when acting on furcellaran. The HPLC and LC–MS results first implied that OUC-FaKC16A and OUC-FaKC16Q could degrade furcellaran and produce desulfated N*κ*COSs, shown to be active also on a substrate different from *κ*-carrageenan. Furthermore, the specific structures of desulfated N*κ*COSs were determined with the help of the ADAG OUC-FaBC129A and combining the MS and HPLC results. We know that the main furcellaran hydrolysates of OUC-FaKC16A and OUC-FaKC16Q were DA-G-(DA-G4S)2 and DA-G-DA-G4S, respectively. The *β*-carrageenase belonging to GH16_13 family was first found in *Paraglaciecola hydrolytica* S66^T^ exhibiting hydrolysis ability toward furcellaran (Schultz-Johansen et al. 2018). Recently, another GH16_13 carrageenase Cgbk16A_Wf from *Wenyingzhuangia fucanilytica* CZ1127 was also identified. It showed the function to hydrolyze furcellaran producing (DA-G4S)_2_-DA-G as the main product (Cao et al. 2021b), which is different from the products of OUC-FaKC16A and OUC-FaKC16Q. Interestingly, these two latter enzymes exhibited a high activity to hydrolyze furcellaran, as well as the ability to produce desulfated N*κ*COSs with specific structures but different DPs. This highlights their significance as a biocatalyst applicable in the preparation of desulfated N*κ*COSs with new structures from the hydrolysis of furcellaran.

## Conclusion

This research constructed the OUC-FaKC16Q based on the N*κ*4-producing *κ*-carrageenase OUC-FaKC16A by truncating the C-terminal noncatalytic FaPorS domain responsible for binding *κ*-carrageenan. Although it demonstrated negative impacts on catalytic efficiency and stability, the most significant result was that this truncation enabled OUC-FaKC16Q to carry out complete hydrolysis of N*κ*4 to N*κ*2. Therefore, an N*κ*2-producing *κ*-carrageenase with high reaction temperature and good thermostability was obtained, further expanding the product spectrum that could be obtained from OUC-FaKC16A. From structure modeling, the OUC-FaKC16Q may form a smaller catalytic channel to accommodate N*κ*4. Further molecular docking indicated OUC-FaKC16Q was more stable than OUC-FaKC16A in disassociating N*κ*4. Another reason for the change of the OUC-FaKC16A product may be the carrageenan-binding capacity of the PorS domain, making it more difficult for the catalytic domain to separate from the substrate after completion of one hydrolysis. So, after truncating the PorS domain, the hydrolysis process accelerated to produce more N*κ*2 products. Besides, their degradation modes toward furcellaran, the main hydrolysates were described. In general, the research herein conducted demonstrated the application potential of *κ*-carrageenase OUC-FaKC16A for preparing oligosaccharides with specific DP and structure, including N*κ*2, N*κ*4, DA-G-(DA-G4S)_2_, and DA-G-DA-G4S, proved to be possible.

## Materials and methods

### Materials

The plasmid pCold for target protein expression was modified from pCold-SUMO by truncating its SUMO tag, achieved via PCR. The *Escherichia coli* DH5*α* for recombinant plasmid construction was from Tsingke Biotechnology Co., Ltd. (Beijing, China). The *E. coli* RTS BL21(DE3) for target protein production was from HaiGene (Qingdao, China). The *κ*-carrageenan and furcellaran used for product determination were from Sigma-Aldrich and Carbosynth (Oxford, England), respectively. The *κ*-carrageenan oligosaccharides standards with DPs of 2, 3, 4, 5, and 8 were from Bz Oligo Biotech (Qingdao, China). The Luria–Bertani liquid medium was used for engineered bacterial growth that composed of 0.5% (w/v) yeast extract, 1% (w/v) tryptone, and 1% (w/v) NaCl.

### Sequence analyses

The conserved domain of OUC-FaKC16A and other enzymes collected in this study were analyzed by the Pfam online database (https://pfam.xfam.org/) (Mistry et al. 2021). Multiple sequence alignment was performed using ESPript 3.0 (http://espript.ibcp.fr/ESPript/ESPript/). Phylogenetic trees were constructed by MEGA 6.0.

### Enzyme expression and purification

The genes encoding OUC-FaKC16A (GenBank number: MCG9793720.1) without the N-terminal signal peptide (SP), OUC-FaKC16Q (Q22-Q325), and FaPorS (L326-Q398) were amplified from the genomic DNA of *F. algicola* using the primers listed in Supplementary Table S1. All the primers used in this study were synthesized by Tsingke Biotechnology Co., Ltd. (Beijing, China). Meanwhile, the pCold plasmid was also linearized by PCR using the primer pColdF/pColdR (Supplementary Table S1). Subsequently, the PCR products of the target genes and the linearized plasmid were linked via ClonExpress® Ultra One Step Cloning Kit (Vazyme, China), then transferred into *E. coli* DH5*α* to obtain the recombinant expression plasmids harboring the target genes. After successful sequencing, the recombinant plasmids were transformed into *E. coli* RTS BL21(DE3) for protein expression.

Engineered strains were first grown at LB liquid medium containing 0.5 mg/mL L-arabinose, 100 μg/mL ampicillin, and 17 μg/mL chloramphenicol, at 37 °C, 200 r/min. A 2 ng/mL tetracycline was added to each flask when the OD600 reached 0.3. Culturing continued until OD600 reached 0.6. The isopropyl *β*-D-thiogalactoside with a final concentration of 0.1 mmol/L was added and decreased the culture temperature to 16 °C for 24 h to induce the protein expression.

After fermentation, cells were collected using centrifugation at 8000 r/min and 4 °C for 15 min, resuspended with ultrapure water and subsequently disrupted by ultrasonic wave. Cellular debris was removed by centrifugation at 8000 r/min and 4 °C for 15 min and the supernatant containing the crude enzyme was purified by Ni^2+^-NTA affinity chromatography. Enzyme purity was checked using 10% SDS-PAGE, and enzyme concentrations determined using Coomassie brilliant blue G250 (Solarbio, China) with BAS as the standard.

### Activity assays

The hydrolytic activities of OUC-FaKC16A and OUC-FaKC16Q toward *κ*-carrageenan or furcellaran were determined using DNS method. In short, a total 200 μL reaction system contained 3 mg/mL *κ*-carrageenan or furcellaran, 20 pure enzymes (OUC-FaKC16A, OUC-FaHC16Q, and FaPorS), and 20 mmol/L Tris-HCl buffer, pH 7.0, at 60 °C for 20 min. To this solution, 300 μL DNS (Solarbio, China) was added and immediately boiled for 5 min. After cooling, 200 μL of the liquid was used to detect its absorbance at 540 nm. One unit of enzymatic activity (U) was defined as the amount of enzyme required to obtain 1 μmol of reducing sugar per min from degrading *κ*-carrageenan or furcellaran. The D-Gal was used as the standard for quantification.

### Binding function analysis of FaPorS

In a 1.5 mL centrifugal tube, a total of 200 μL composed of 0.1 mg FaPorS, 2 mg *κ*-carrageenan powder, and 20 mmol/L Tris-HCl buffer, pH 7.5, was incubated at 4 °C and 40 r/min for 2 h. After incubation, the suspension was centrifuged at 12000 r/min and 4 °C for 5 min. Then the supernatant was transferred to another clean tube, while the precipitate was used to resuspend in water. SDS-PAGE was further performed to analyze the bound and unbound proteins.

SEM was used to analyze the surfaces of *κ*-carrageenan after BSA and FaPorS treatment. To this end, 4 mg/mL *κ*-carrageenan was dissolved in 20 mmol/L Tris–HCl buffer (pH 7.5) and incubated with BSA or FaPorS at 37 °C and 200 r/min for 24 h.

### Biochemical characterization of OUC-FaKC16Q

The optimum reaction temperature of OUC-FaKC16Q was decided by setting different temperatures (30, 40, 50, 60, 70, and 80 °C) at 20 mmol/L Tris–HCl buffer (pH 7.0) for 30 min. The optimum reaction pH of OUC-FaKC16Q was determined using 20 mmol/L citric acid–Na_2_HPO_4_ buffer (pH 3.0 to 6.0), 20 mmol/L NaH_2_PO_4_–Na_2_HPO_4_ buffer (pH 6.0 to 7.0), 20 mmol/L Tris–HCl buffer (pH 7.0 to 9.0) and 20 mmol/L glycine–NaOH buffer (pH 9.0 to 10.0) at 60 °C for 30 min. After reactions were completed, the released reducing sugars were determined using the DNS method described above. All measurements were performed in triplicate.

To determine temperature stability, pure OUC-FaKC16A and OUC-FaKC16Q were incubated at 30, 40, 50, 60, and 70 °C for 1 h to measure their residual activity. Hereafter, the longer incubation time at 30 and 40 °C was performed to further compare their thermostability. The influence of different metal ions on the activities of OUC-FaKC16A and OUC-FaKC16Q was tested using a concentration of 5 mmol/L of the desired metal ion (Ba^2+^, Co^2+^, Fe^2+^, K^+^, Mg^2+^, Na^+^, Zn^2+^, Ni^2+^, Na_2_EDTA, and SDS). All measurements were performed in triplicate.

To calculate the *K*_m_ and *k*_cat_ of OUC-FaKC16A and OUC-FaKC16Q, the *κ*-carrageenan solutions with different concentrations from 0 g/L to 3 g/L were incubated with pure OUC-FaKC16A or OUC-FaKC16Q at 60 °C and pH 7.0 or 9.0 for 20 min. After the reaction, the released reducing sugars were quantified by DNS. Next, the values of *K*_m_ and *k*_cat_ were determined using the Lineweaver–Burk equation.

### Degradation mode of OUC-FaKC16Q towards *κ*-carrageenan

Samples of 300 μL reaction volume containing 3 g/L *κ*-carrageenan, 1 U OUC-FaKC16Q, and 20 mmol/L pH 7.0 Tris–HCl buffer were performed at 60 ℃ for different times. The samples were further analyzed using HPLC equipped with Superdex 30 10/300 gel filtration column (GE Health, Marlborough, MA, USA) as previously reported (Jiang et al. 2022).

The reaction products from hydrolyzing *κ*3, *κ*5, and N*κ*8 were also detected using the HPLC method described above.

### Homology modeling, and molecular docking

The three-dimensional structures of OUC-FaKC16A and OUC-FaKC16Q were predicted by Phyre2 online tool via Intensive modeling mode (Kelley et al. 2015). The simulated structures were further evaluated by VERIFY3D (Eisenberg et al. 1997) to demonstrate their reliability. The PDB files of OUC-KC16A and OUC-FaKC16Q were further processed by AutoDockTools to produce PDBQT file as the docking receptor (Morris et al. 2009). The 3D structure of N*κ*4 was obtained from the PDB file of PcCgkA_GH16-E168D_ (PDB number: 5OCQ). The docking of receptors (OUC-FaKC16A and OUC-FaKC16Q) with N*κ*4 was then performed using AutoDock Vina (Trott and Olson 2010). The docking conformations were analyzed using PYMOL (https://pymol.org/2/) and Discovery Studio 4.5 visualizer.

### Products analyses of OUC-FaKC16A and OUC-FaKC16Q from the hydrolysis of furcellaran

Samples of 300 μL reaction volume containing 3 g/L furcellaran, 1 U OUC-FaKC16A or OUC-FaKC16Q, and 20 mmol/L glycine-NaOH (pH 9.0) or Tris-HCl buffer (pH 7.0) were incubated at 60 °C for different times (10 min, 0.5, 1, 2, and 8 h). These samples were detected using the HPLC method described above. Meanwhile, the 8-h products were also analyzed using MS with electrospray ionization, the detailed operation is as previously reported (Jiang et al. 2022).

Besides, LC–MS was also used to analyze hydrolytic products. Analyses were performed with an ultra-performance LC unit (Dionex Ultimate 3000, Thermo Fisher Scientific, U.S.A.) equipped with an Acquity UPSEC BEH 125 SEC column (4.6 × 150 mm, Waters, Milford, U.S.A.) and connected to a Q-Exactive Orbitrap MS (Thermo Fisher Scientific, U.S.A.) The analysis process was conducted at room temperature using 20% (v/v) methanol containing 10 mmol/L ammonium acetate as the mobile phase at a flow rate of 0.2 mL/min (Zhang et al. 2020).

## Supplementary Information

Below is the link to the electronic supplementary material.

Supplementary file1 (DOC 20159 KB)

## Data Availability

The protein sequence of OUC-FaKC16A has been deposited to GenBank with accession number: MCG9793720.1.
