# Supplementary material for: Expanding the application range of the κ‑carrageenase OUC-FaKC16A when preparing oligosaccharides from κ-carrageenan and furcellaran
Source: Mar Life Sci Technol. 2023 Jul 12;5(3):387–99. doi: 10.1007/s42995-023-00181-2 (PMC10449746; doi:10.1007/s42995-023-00181-2)
Supplement: Supplementary file 1 — Supplementary file1 (DOC 20159 KB) [file 42995_2023_181_MOESM1_ESM.doc]

**Supplement materials**

**Expanding the application range of the *κ*‑carrageenase OUC-FaKC16A in preparing oligosaccharides from *κ*-carrageenan and furcellaran**

Chengcheng Jiang^1, 3^, Francesco Secundo^4^, Xiangzhao Mao^1,2,3*^

^1^ College of Food Science and Engineering, Ocean University of China, Qingdao 266003, China

^2^ Laboratory for Marine Drugs and Bioproducts of Qingdao National Laboratory for Marine Science and Technology, Qingdao 266237, China

^3^ Key Laboratory for Biological Processing of Aquatic Products, China National Light Industry, Qingdao 266237, China

^4^ Istituto di Scienze e Tecnologie Chimiche "Giulio Natta", Consiglio Nazionale delle Ricerche, via Mario Bianco 9, 20131 Milan, Italy

^*^Corresponding author: Xiangzhao Mao

Address: College of Food Science and Engineering, Ocean University of China, Qingdao 266003, China

Tel.: +86-532-82032660

Fax: +86-532-82032272

E-mail: [xzhmao@ouc.edu.cn](mailto:xzhmao@ouc.edu.cn)


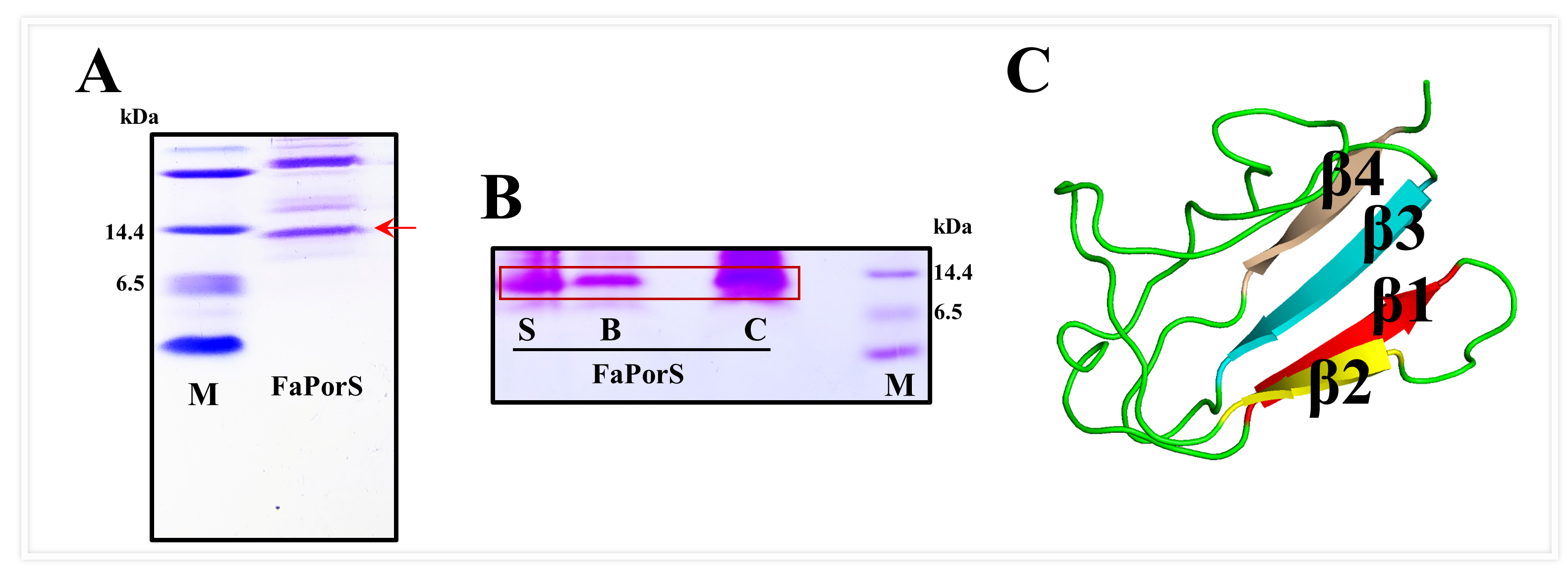


**Fig. S1**. SDS-PAGE analysis of the purity of FaPorS (A) and to evaluate the binding function of FaPorS towards *κ*-carrageenan (B). Three-dimensional structure of FaPorS predicted by Phyre2 online tool via Intensive modelling mode (C).


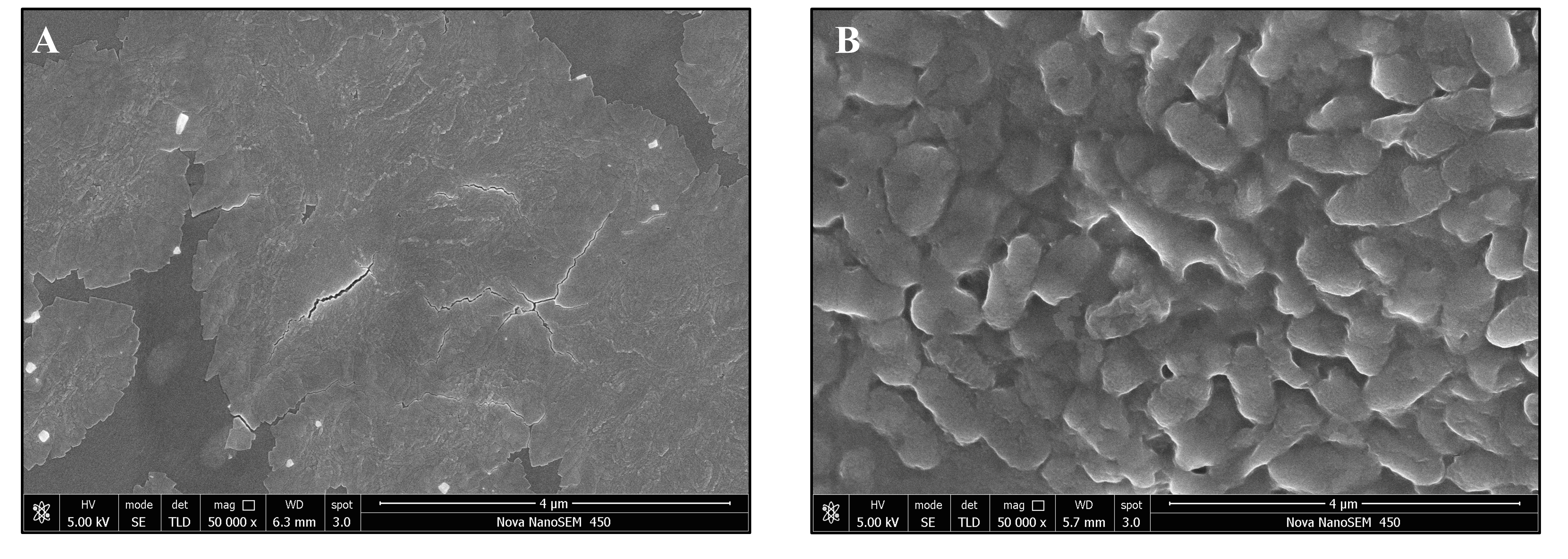


**Fig. S2**. Scanning electron microscopy (SEM) analyses of the surfaces of *κ*-carrageenan with BSA (A) and FaPorS (B) treatment.


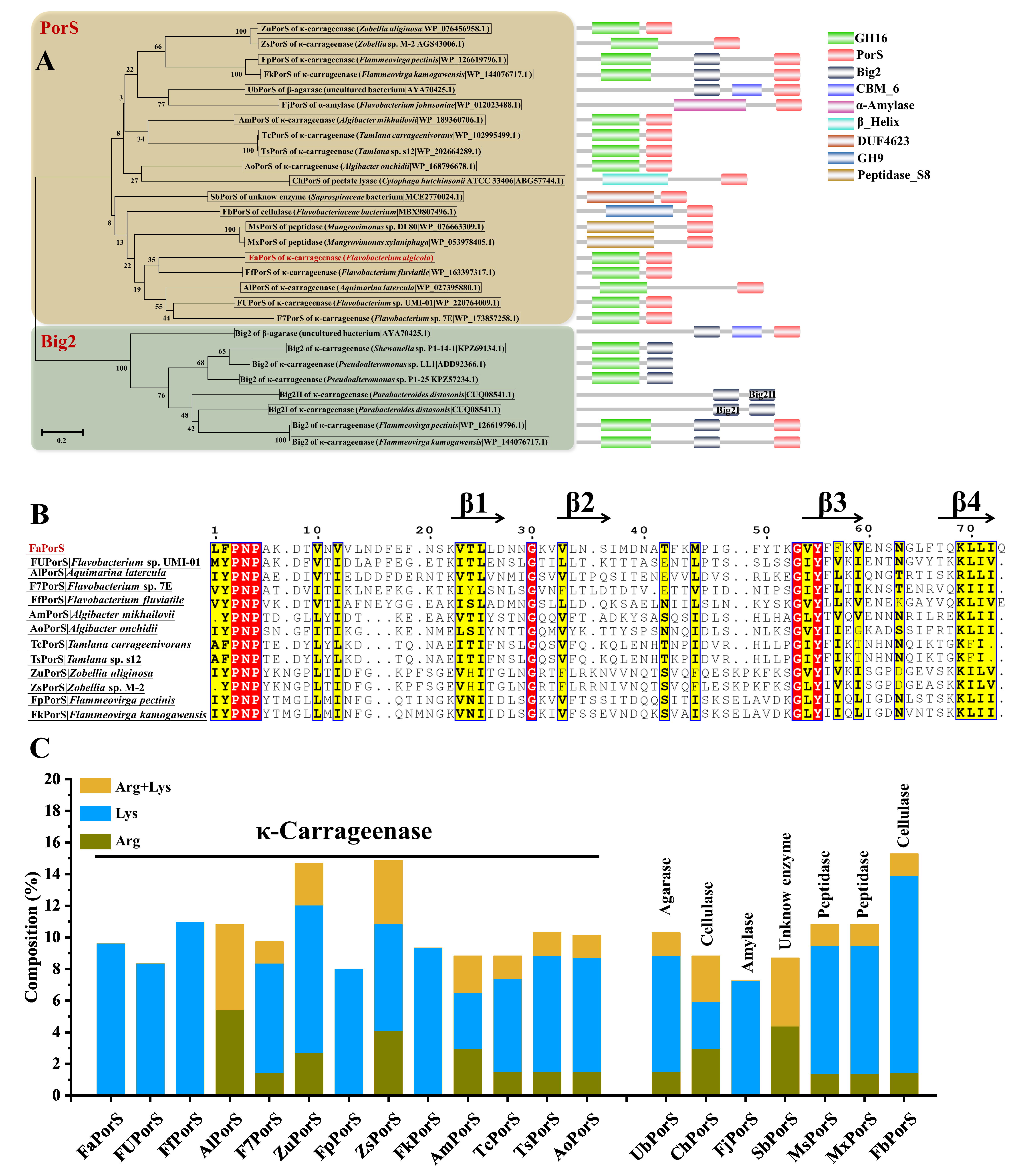


**Fig. S3**. Phylogenetic relationship of PorS domain with several Ig-like domains (A). Sequence alignment of the PorS domains (B). Positively charged amino acid (Arg and Lys) compositions of the PorS domain from different hydrolases (C).


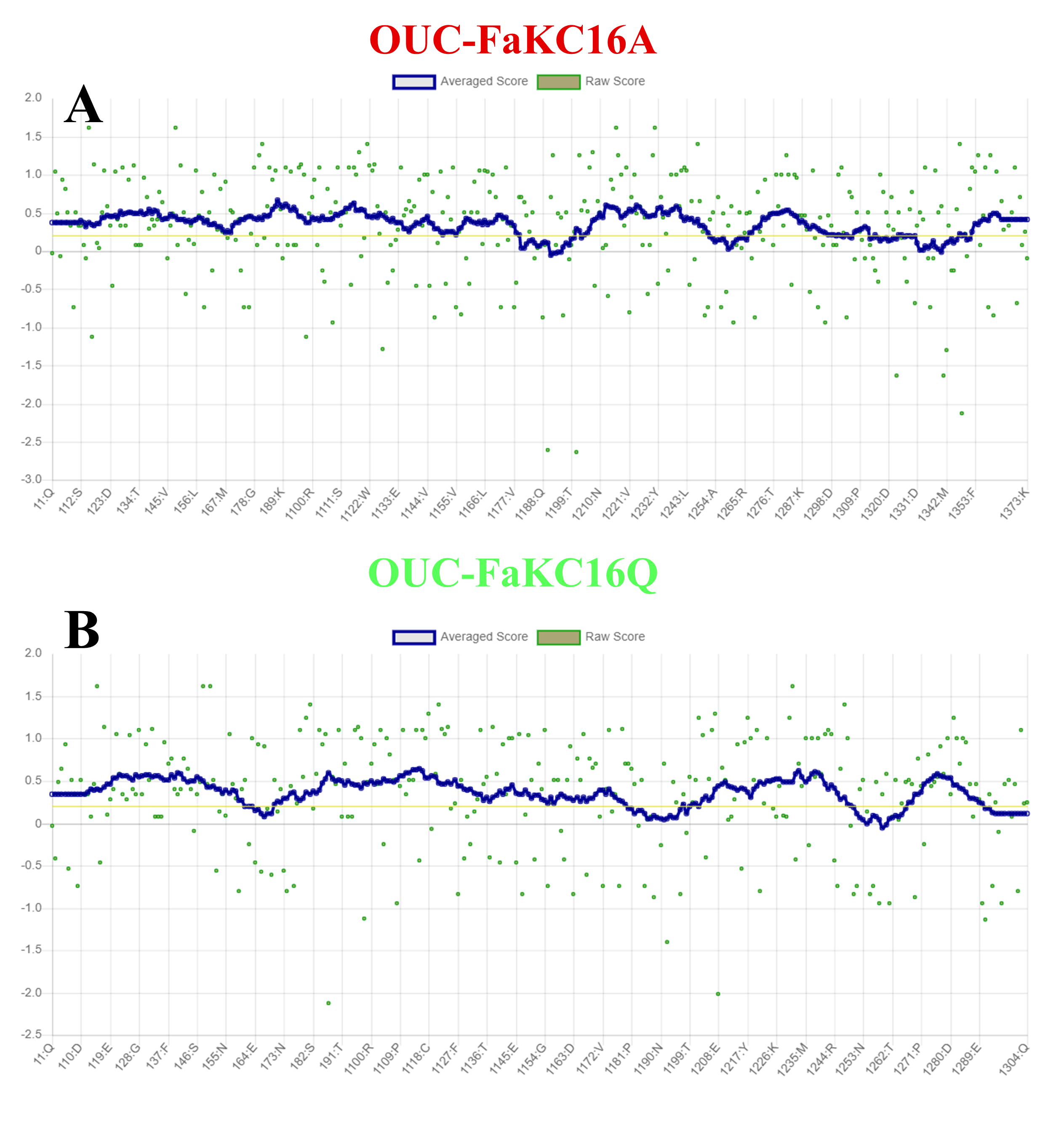


**Fig. S4** VERIFY3D output result of predicted three-dimensional structures of OUC-FaKC16A (A) and OUC-FaKC16Q (B) for evaluating their reliability.


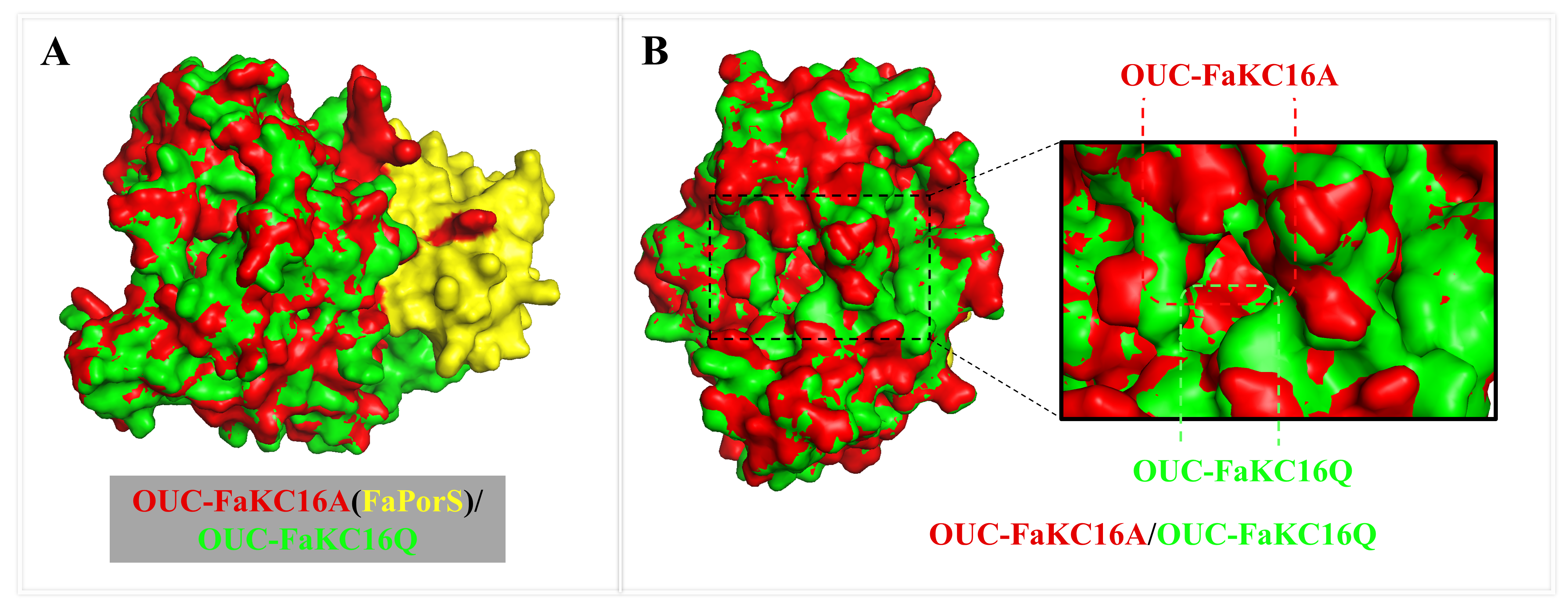


**Fig. S5.** Comparison of the three-dimensional structure of OUC-FaKC16A and OUC-FaKC16Q showed as surface style. Red and green colors indicated the three-dimensional structure of OUC-FaKC16A and OUC-FaKC16Q, respectively. Yellow color indicated the FaPorS in OUC-FaKC16A.


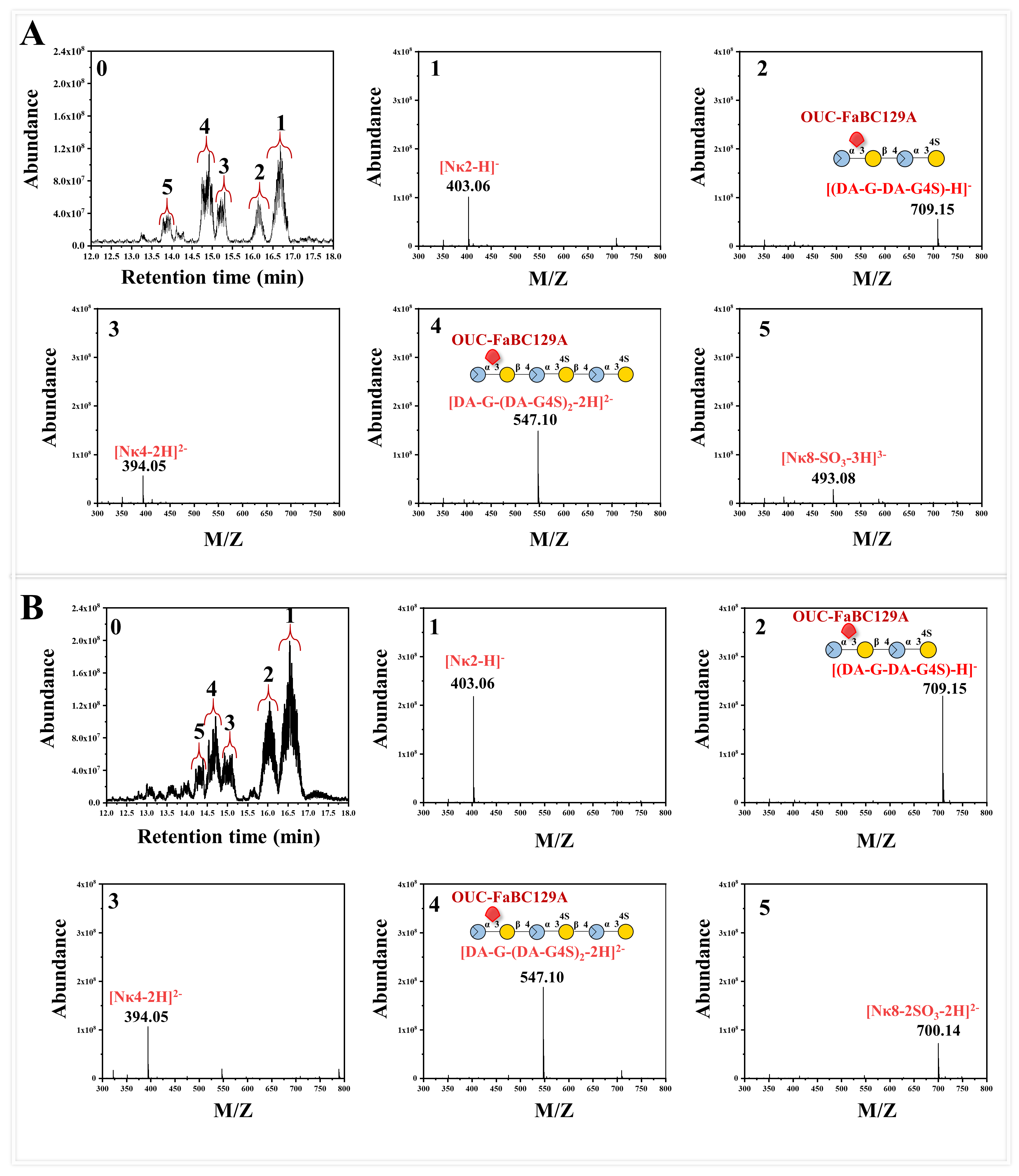


**Fig. S6** LC-MS analyses of the products of the hydrolysis of furcellaran using OUC-FaCK16A (A) and OUC-FaKC16Q (B)

**Table S1** The primers used in this study.

| Primer | Sequence (5'-3') | Usage |
| --- | --- | --- |
| OUC-FaKC16A/Q-F | ATGGCGCAACATGCACCTATAATAAATGATGTAG | Cloning gene *ouc-FaKC16A* to ligate into linearized pCold |
| OUC-FaKC16A/PorS-R | TCAGTGGTGGTGGTGGTGGTGCTCGAGTGCGGCCGCAAGCTCTGTATTAAAAGTTTTTGGG |
| OUC-FaKC16A/Q-F | ATGGCGCAACATGCACCTATAATAAATGATGTAG | Cloning gene *ouc-FaKC16Q* to ligate into linearized pCold |
| OUC-FaKC16Q-R | TCAGTGGTGGTGGTGGTGGTGCTCGAGTGCGGCCGCAAGCTCTTGCCTTCCTACTTTTTTATC |
| PorS-F | cggcggtggcggtagcggtCTCTTTCCTAATCCTGC | Cloning gene *porS* to ligate into linearized pCold |
| OUC-FaKC16A/PorS-R | TCAGTGGTGGTGGTGGTGGTGCTCGAGTGCGGCCGCAAGCTCTGTATTAAAAGTTTTTGGG |
| pCold-F | CCGCACTCGAGCACCACCACCACCACCACTGAaagcttgtcgacctgcag | To linearize the plasmid pCold-SUMO without SUMO domain for linking target genes |
| pCold-R | aatgggtcgcggatccGCAAATGGAACTCCACC |
| R265A-F | GTGCCTTTTGTACAATTTG | To construct OUC-FaKC16QR265A mutant |
| R265A-R | CAAATTGTACAAAAGGCACCGCAAGTCCTAAAGAAACCGTAAC |
